# Correlation between visceral fat area and cardiac valve calcification in hemodialysis patients

**DOI:** 10.3389/fcvm.2025.1574649

**Published:** 2025-04-10

**Authors:** Xiaoqi Wang, Dan Yuan, Feng Shao, Jingjing Zhou, Xiao Zhang, Zhongxin Li

**Affiliations:** Beijing Luhe Hospital, Capital Medical University, Beijing, China

**Keywords:** maintenance hemodialysis, bioelectrical impedance analysis, visceral fat area, cardiac valve calcification, heart

## Abstract

**Objective:**

To investigate the relationship between Visceral Fat Area (VFA) and cardiac valve calcification (CVC) in Maintenance Hemodialysis (MHD) patients.

**Methods:**

This cross-sectional study included MHD patients enrolled at our hospital between July 2023 and February 2024. Body composition analysis was performed on recruited patients. According to echocardiography results, the participants were classified into 2 groups. We then compared their clinical characteristics and identified independent factors influencing CVC through multivariate logistic regression. The ROC curve was employed to assess the ability of influencing factors to predict CVC in MHD patients.

**Results:**

There are 154 MHD patients were recruited, including 76 with CVC and 78 without CVC. Significant differences were observed between CVC and non-CVC participants in age, the proportion of diabetic nephropathy, the proportion of diabetes mellitus, the levels of Hs-CRP, fasting blood glucose, blood phosphorus, iPTH, HDL-C and VFA (*P* < 0.05). Advanced age, diabetes, increased VFA and iPTH all have the ability to predict individuals with CVC in MHD patients based on Multivariate Logistic regression. ROC curve indicated that VFA could accurately identify individuals with CVC among MHD patients (AUC = 0.713). When age, diabetes, iPTH, and VFA were combined for predicting CVC, the AUC was 0.776 (*P* < 0.01), which was greater than any single indicator.

**Conclusions:**

For MHD patients, increased VFA may serve as a potential marker for detecting CVC and can assist in clinical decision-making.

## 1 Introduction

Cardiac valve calcification (CVC) is linked to a higher risk of cardiovascular events and overall mortality in maintenance hemodialysis (MHD) patients, with its severity directly correlating with these outcomes (1). Multiple studies have demonstrated that CVC in MHD patients is linked to age, comorbid diabetes mellitus (2), disordered calcium-phosphorus metabolism (3), systemic microinflammatory states (4), and conditions such as malnutrition or sarcopenia (5). Metabolic syndrome and obesity are highly prevalent among MHD patients, and dyslipidemia has been linked to the occurrence and progression of CVC (6). Therefore, improving the evaluation of nutritional and metabolic status to guide clinical management has gained increasing attention.

Visceral fat area (VFA) is a novel indicator for evaluating dyslipidemia. Compared to traditional obesity measures such as body mass index (BMI), body weight, and waist-to-hip ratio, elevated VFA has been linked to arterial stiffness and coronary artery calcification in patients with diabetes (7). Furthermore, it is closely linked to the occurrence of cardiovascular disease, the development of chronic kidney disease, and higher all-cause mortality (8, 9). However, the relationship between VFA and CVC in MHD patients has yet to be fully understood.

In this context, the current research seeks to explore the relationship between VFA levels and CVC in MHD patients and to analyze contributing factors for CVC as well as the potential of VFA as a predictor for CVC in this population.

## 2 Material and methods

### 2.1 Study participants

**Figure 1:**
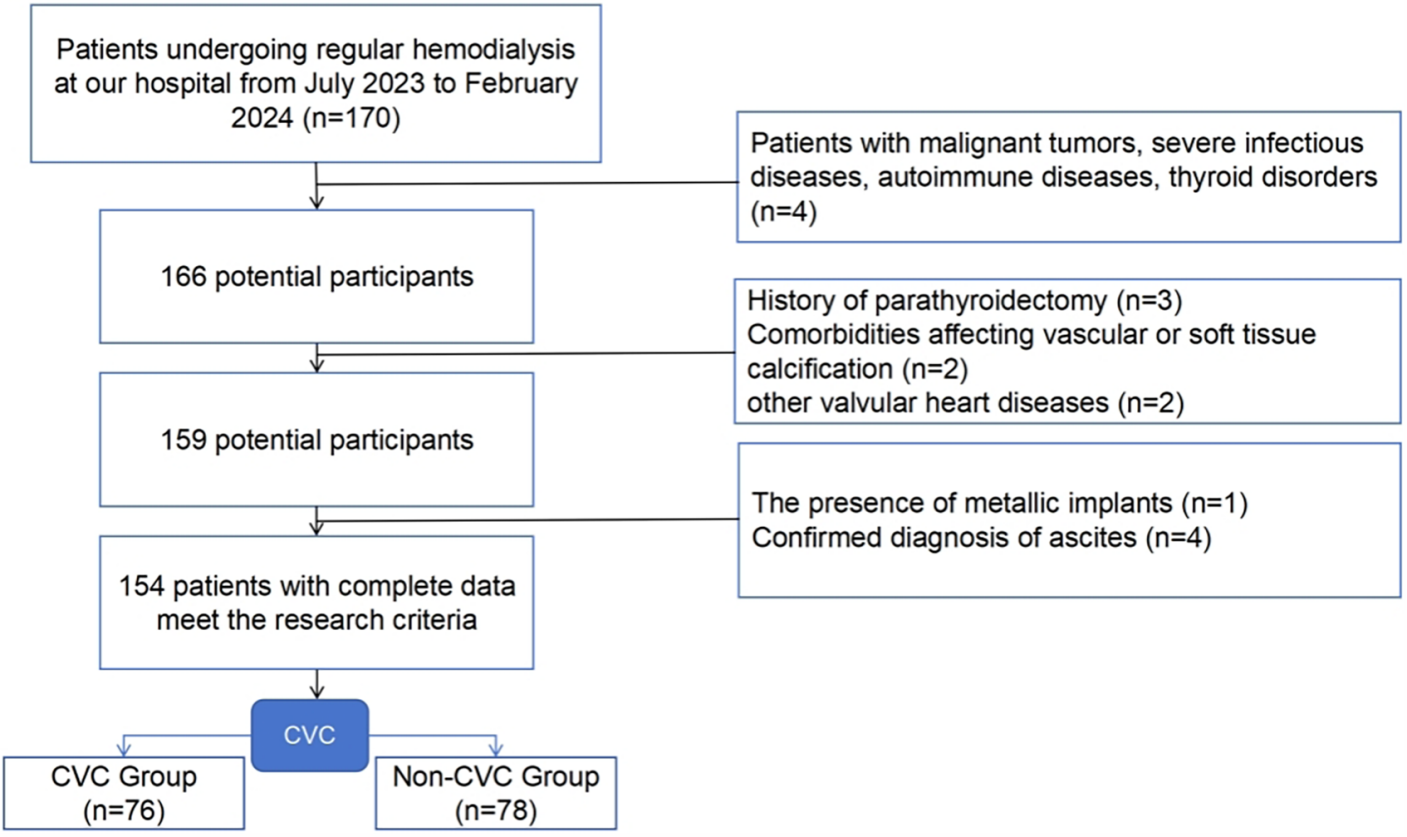
Flowchart of inclusion and exclusion of participants.

Participants undergoing regular hemodialysis at our hospital from July 2023 to February 2024 were enrolled in this research. Body composition was evaluated through bioelectrical impedance analysis (BIA). The inclusion criteria included: (1) age of 18 years or older; (2) receiving hemodialysis 3 times weekly, with each session lasting 3.5–4 h, a blood flow rate of 200–300 ml/min, and dialysis vintage ≥3 months; (3) the ability to stand independently and cooperate for body composition analysis; and (4) willingness to undergo a complete echocardiography examination. The exclusion criteria included: (1) patients with malignant tumors, severe infectious diseases, autoimmune diseases, thyroid disorders, etc.; (2) history of parathyroidectomy; (3) comorbidities affecting vascular or soft tissue calcification, such as amyloidosis and multiple myeloma; (4) other valvular heart diseases, including rheumatic heart disease, infective endocarditis, and congenital heart conditions; (5) presence of metallic implants; and (6) confirmed diagnosis of ascites. Informed consent was obtained from all participants, and the study received approval from the Beijing Luhe Hospital ethics committee (Approval Number: 2023-LHKY-012-02). The inclusion and exclusion flowchart is as follows (Figure 1).

### 2.2 Study methods

#### 2.2.1 General patient data and laboratory tests

General data were gathered for all participants, including age, sex, dialysis vintage, primary disease, diabetic retinopathy (classified as proliferative or non-proliferative), and comorbidities (hypertension, stroke, ischemic heart disease, peripheral vascular disease, smoking history). Height and weight were recorded, and body mass index (BMI) was calculated using the formula: BMI = weight (kg)/height^2^ (m^2^). Vascular access type (arteriovenous fistula vs. central venous catheter) was documented based on clinical records. Laboratory test results closest to the date of echocardiography were retrieved from the hospital's integrated hemodialysis information system. The parameters collected included white blood cell count (WBC), hemoglobin (HB), high-sensitivity C-reactive protein (Hs-CRP), serum albumin, blood glucose, serum creatinine, blood urea nitrogen, serum potassium, sodium, calcium, phosphorus, uric acid, total cholesterol (TC), triglycerides (TG), low-density lipoprotein cholesterol (LDL-C), high-density lipoprotein cholesterol (HDL-C), intact parathyroid hormone (iPTH), and brain natriuretic peptide (BNP).

#### 2.2.2 VFA measurement

VFA measurement was conducted immediately after the completion of dialysis. A body composition analyzer utilizing the BIA method was employed to assess VFA. VFA was assessed non-invasively using the InBody 770 body composition analyzer (Biospace Co., Ltd., Seoul, South Korea; distributed by Baisibei Medical Device Trading Co., Ltd., Shanghai, China). This system employs multi-frequency BIA technology, which applies low-level alternating currents at six discrete frequencies (1 kHz, 5 kHz, 50 kHz, 250 kHz, 500 kHz, and 1 MHz) to measure tissue resistance. Fat, muscle, and body water exhibit distinct conductive properties, enabling the device to differentiate their proportions and calculate VFA.

During the procedure, participants stood barefoot on the analyzer's platform, aligning their soles with four integrated foot electrodes. Handheld electrodes were grasped firmly, with thumbs and palms in full contact, while arms were positioned at approximately 30° from the torso to prevent limb-trunk contact. Participants maintained a static posture with elbows fully extended for 60 s. All measurements were performed in a temperature-controlled room (25 ± 1 °C) 2 h post-dialysis, following a standardized protocol to minimize variability.

#### 2.2.3 Cardiac valve calcification assessment

CVC was assessed by two experienced ultrasonographers using cardiac echocardiography, following the guidelines recommended by the Kidney Disease: Improving Global Outcomes (KDIGO) organization (10). Multiplane echocardiographic imaging was conducted to evaluate echogenicity of cardiac valve tissues. The diagnostic criteria for CVC are the presence of high echogenic signals greater than 1 mm on the aortic valve, mitral valve, or mitral annulus. According to the presence or absence of CVC, MHD patients were categorized into two groups.

### 2.3 Statistical methods

Statistical analyses were performed using SPSS 22.0 (IBM Corp., Armonk, NY, USA) for Windows. Continuous variables following a normal distribution were represented as mean ± SD. Categorical variables were described in terms of frequency and percentage. Independent sample *t*-tests were applied for continuous variables with normal distribution, while non-normally distributed continuous variables were analyzed using the Mann–Whitney *U* test for comparison between the two groups. The chi-square (*χ*^2^) test was applied to evaluate categorical variables. Logistic regression analysis was employed to identify independent risk factors associated with CVC. Multicollinearity among variables was assessed using variance inflation factors (VIF). All variables showed VIF values <5 (range: 1.12–3.89), indicating no significant multicollinearity. The predictive capacity of VFA for CVC was assessed through ROC curve. Statistical significance was defined as *P* < 0.05.

## 3 Results

### 3.1 Comparison of basic clinical characteristics of enrolled participants

This research included 154 MHD patients, with ages ranging from 24 to 85 years (median age: 60 years). Of these, 97 participants (63.0%) were male, while 57 participants (37.0%) were female. CVC was present in 49.4% of patients, and they were placed in the CVC group.

The two groups were similar regarding gender, hypertension, dialysis vintage, BMI, WBC, HB, serum albumin, serum creatinine, blood urea nitrogen, serum potassium, sodium, calcium, uric acid, TC, TG, LDL-C, diabetic retinopathy, vascular access type, stroke history, ischemic heart disease, peripheral vascular disease, smoking history, or BNP (*P* > 0.05). However, the CVC group demonstrated significantly higher age, prevalence of diabetic nephropathy, comorbid diabetes, Hs-CRP, fasting blood glucose, serum phosphorus, iPTH, and VFA compared to the non-CVC group. Conversely, HDL-C levels were significantly lower in the CVC group compared to non-CVC group (*P* < 0.05) (Table 1).

**Table 1:** Clinical characteristics.

Baseline characteristics	CVC group (*n* = 76)	Non-CVC group (*n* = 78)	*F*/*χ*2/*H*	*P*
Age (years)	63 (51.25, 69)	56 (44.75, 64.25)	3.14	<0.01**
Gender (Male/Female)	47/29	50/28	0.08	0.77
Diabetic nephropathy (*n*, %)	33 (43.4%)	16 (20.5%)	9.31	<0.01**
Comorbid diabetes (*n*, %)	45 (59.2%)	26 (33.3%)	10.34	<0.01*
Comorbid hypertension (*n*, %)	70 (91.1%)	72 (92.3%)	0.02	0.96
Stroke history (*n*, %)	9 (11.8%)	7 (9.0%)	0.38	0.54
Ischemic heart disease (*n*, %)	15 (19.7%)	12 (15.4%)	0.57	0.45
Peripheral vascular disease (*n*, %)	6 (7.9%)	5 (6.4%)	0.12	0.73
Smoking history (*n*, %)	22 (28.9%)	20 (25.6%)	0.22	0.64
Diabetic retinopathy			1.24	0.54
– Proliferative (*n*, %)	12 (15.8%)	10 (12.8%)	—	—
– Non-proliferative (*n*, %)	16 (21.1%)	14 (17.9%)	—	—
Vascular access type			0.11	0.74
– AV fistula (*n*, %)	65 (85.5%)	68 (87.2%)	—	—
– Central venous catheter (*n*, %)	11 (14.5%)	10 (12.8%)	—	—
Dialysis vintage (months)	60.93 ± 42.26	56.03 ± 51.50	3.15	0.52
BMI (kg/m2)	24.79 ± 3.83	23.84 ± 3.28	1.81	0.10
WBC (×109/L)	7.67 ± 1.94	7.2 ± 1.8	0.37	0.13
HB (g/L)	112.99 ± 11.18	112.36 ± 11.42	0.03	0.73
Hs-CRP (mg/L)	9.55 ± 15.94	5.35 ± 7.39	9.11	0.04*
ALB (g/L)	40.23 ± 3.75	41.18 ± 2.99	1.92	0.08
GLU (mmol/L)	7.47 (6.14, 10.77)	6.25 (5.33, 7.55)	3.24	<0.01**
Scr (μmol/L)	868.53 ± 229.1	929.35 ± 245.88	0.72	0.12
BUN (mmol/L)	23.02 ± 5.2	23.69 ± 5.52	0.07	0.44
K (mmol/L)	4.99 ± 0.78	4.87 ± 0.73	0.60	0.33
Na (mmol/L)	134.7 ± 15.89	136.77 ± 2.76	2.11	0.26
Ca (mmol/L)	2.21 ± 0.17	2.2 ± 0.18	1.11	0.90
P (mmol/L)	1.98 (1.47, 2.46)	1.74 (1.41, 2.04)	2.65	0.01*
iPTH (pg/L)	308.88 ± 191.41	228.68 ± 146.4	7.82	0.01*
UA (μmol/L)	421.78 ± 85.29	404.13 ± 82.12	0.98	0.19
TC (mmol/L)	4.37 ± 0.91	4.22 ± 0.95	0.01	0.32
TG (mmol/L)	2.19 ± 1.57	1.89 ± 1.08	6.79	0.17
LDL-C (mmol/L)	2.64 ± 0.68	2.45 ± 0.66	0.37	0.08
HDL-C (mmol/L)	0.95 ± 0.24	1.07 ± 0.4	8.27	0.03*
BNP (pg/ml)	512.98 ± 811.17	429.87 ± 575.9	1.21	0.46
VFA (cm2)	97.55 (88.15, 110.33)	80.75 (58.85, 93.6)	4.56	<0.01**

* Indicates *P* < 0.05.

** Indicates *P* < 0.01.

### 3.2 Analysis of influencing factors for CVC in MHD patients

CVC status in MHD patients (present = 1, absent = 0) was set as the dependent factor. Variables showing significant differences between groups (*P* < 0.05) were incorporated into a multivariate logistic regression model. Considering the clinical similarity in significance between fasting blood glucose and diabetes status, this variable was excluded. Ultimately, nine variables were included as independent variables: age, primary disease (diabetic nephropathy or not), comorbid diabetes, Hs-CRP, serum phosphorus, iPTH, HDL-C, and VFA levels.

The multivariate analysis revealed that advanced age (OR = 1.035, *P* = 0.027), comorbid diabetes (OR = 2.531, *P* = 0.011), elevated iPTH (OR = 1.003, *P* = 0.013), and increased VFA levels (OR = 1.020, *P* = 0.002) were independent predictors for CVC. Other variables, including primary disease (diabetic nephropathy), Hs-CRP, serum phosphorus, HDL-C, and sex, did not show statistically significant associations with CVC in the adjusted model (*P* > 0.05) (Table 2).

**Table 2:** Multivariate logistic regression analysis of factors influencing CVC in MHD patients.

Factor	*β*	S.E.	Wald	Degrees of freedom	*P*	OR	95% CI	
							Lower	Upper
Age	0.034	0.015	4.899	1.000	0.027	1.035	1.004	1.066
Sex (Male)	−0.193	0.302	0.408	1.000	0.523	0.825	0.456	1.492
VFA	0.020	0.007	9.479	1.000	0.002	1.020	1.007	1.034
iPTH	0.003	0.001	6.212	1.000	0.013	1.003	1.001	1.005
Hs-CRP	0.018	0.011	2.647	1.000	0.104	1.018	0.996	1.041
Serum phosphorus	0.205	0.129	2.527	1.000	0.112	1.227	0.953	1.580
HDL-C	−0.621	0.351	3.132	1.000	0.077	0.537	0.270	1.069
Primary disease^a^	0.412	0.288	2.042	1.000	0.153	1.509	0.857	2.658
Comorbid diabetes	0.929	0.366	6.430	1.000	0.011	2.531	1.235	5.189

^a^ Primary disease refers to diabetic nephropathy.

VFA, visceral fat area; iPTH, intact parathyroid hormone.

### 3.3 Predictive performance of VFA for CVC in MHD patients

**Figure 2:**
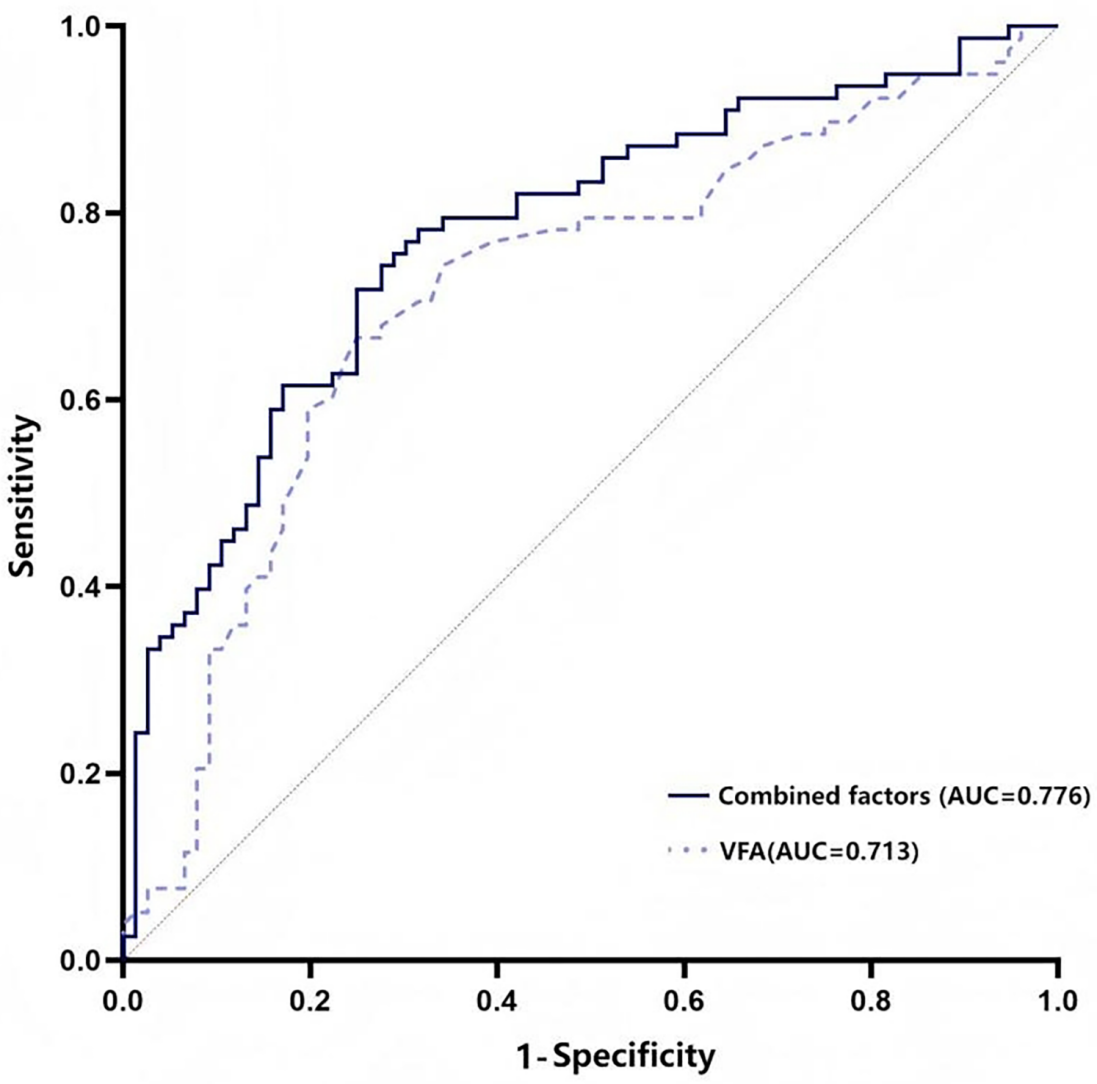
ROC curve of various factors predicting CVC in MHD patients. The combined predictor refers to the integration of the four variables: diabetes status, serum iPTH, age, and VFA level.

The ROC curve revealed that diabetes status, serum iPTH, and age showed certain predictive value for CVC in MHD patients, with area under the curve (AUC) values of 0.629, 0.620, and 0.646, respectively (*P* < 0.01). VFA demonstrated a higher predictive value for CVC, with an AUC of 0.713 (*P* < 0.01), a sensitivity of 75.0%, and a specificity of 66.7%. When diabetes status, serum iPTH, age, and VFA were combined as predictors, the AUC increased to 0.776, which was greater than any single indicator, with a sensitivity of 72.4% and a specificity of 74.4% (*P* < 0.01). Details are presented in Table 3 and Figure 2.

**Table 3:** Predictive performance of factors for CVC in MHD patients.

Factor	AUC	*P*	Sensitivity	Specificity	95% CI	
					Lower	Upper
Comorbid diabetes	0.629	<0.01	0.592	0.667	0.541	0.718
iPTH	0.620	0.010	0.447	0.782	0.532	0.709
Age	0.646	< 0.01	0.566	0.692	0.560	0.733
VFA	0.713	< 0.01	0.750	0.667	0.630	0.796
Combined factors	0.776	< 0.01	0.724	0.744	0.702	0.850

VFA, visceral fat area; iPTH, intact parathyroid hormone.

The combined predictor refers to the integration of the four variables: diabetes status, serum iPTH, age, and VFA level.

## 4 Discussion

CVC is a common complication in MHD patients, contributing to a higher probability of cardiovascular events and overall mortality, thereby negatively impacting patient prognosis. In our research, 48.4% of MHD patients was found to develop CVC. Recent studies have reported a prevalence of CVC ranging from 31.7% to 64.8% (2, 11, 12). The apparent variation in CVC prevalence across different centers may be attributed to differences in the average age and dialysis vintage in the patient cohorts.

BIA was employed to assess VFA in order to investigate its relationship with CVC in MHD patients. While computed tomography (CT) and magnetic resonance imaging (MRI) are regarded as the most precise techniques for assessing VFA (13), their widespread use is limited by high costs and radiation risks. BIA measures resistance values by creating a closed current loop through hand-held electrodes. Since different tissues exhibit different resistances, the fat content within the body can be calculated, and VFA can be derived. BIA is increasingly used in clinical practice due to its advantages, including non-radioactivity, affordability, good accuracy, and high repeatability (14, 15). Recent studies have shown that BIA measurements of VFA correlate well with abdominal CT measurements (r values ranging from 0.6360 to 0.920, *P* < 0.01) (16, 17).

This study identified increased VFA as an independent predictor of CVC in MHD patients (AUC = 0.713). Notably, the BMI of participants in both groups was similar, suggesting that VFA is a more suitable indicator for evaluating the link between nutritional status and prognosis in MHD patients (18). Previous studies have consistently demonstrated that increased visceral fat tissue is an important trigger for the occurrence and development of vascular calcification. For instance, a cross-sectional research from Korea involving 60,938 asymptomatic adults found that visceral fat accumulation was associated with coronary artery calcification and arterial stiffness. Similarly, a study of elderly individuals in China (*n* = 4,068) reported similar findings (19). Another study (*n* = 436) found a correlation between increased VFA and abdominal aortic calcification (20).

In studies involving chronic kidney disease (CKD) patients, increased VFA was also linked to coronary artery calcification (21). Early research from our center revealed a relationship between increased VFA and abdominal aortic calcification in MHD patients (22). Additionally, increased VFA appears to predict cardiovascular events and overall mortality in MHD patients (9).

The mechanism by which increased VFA influences the occurrence of CVC in MHD patients remains inconclusive. Fat tissue is both a terminal organ for energy storage and has endocrine functions (23). Adipose tissue secretes peptides and metabolites, collectively referred to as adipokines, which regulate perivascular inflammation, stimulate osteogenic differentiation, proliferation, and apoptosis of cells, and induce ectopic deposition of calcium and phosphorus, thereby affecting vascular calcification (24, 25). Under conditions of nutritional excess, rapid proliferation and hypertrophy of visceral adipose tissue that outpaces angiogenesis can create a hypoxic microenvironment, which induces the osteogenic transdifferentiation, proliferation, and apoptosis of vascular smooth muscle cells (VSMCs) and endothelial cells (26). Additionally, hypoxia can promote the release of pro-inflammatory adipokines like visfatin, IL-6, and TNF-α from visceral adipocytes, further stimulating macrophage infiltration and inducing osteogenic transdifferentiation of VSMCs and endothelial cells. This exacerbates vascular inflammation and calcification (27).

This study also identified other predictors for CVC in MHD patients, including diabetes, high iPTH, and advanced age, consistent with findings from previous studies (2, 4, 11). Data from the China Dialysis Calcification Study (3) indicate that maintaining calcium, phosphorus, and iPTH concentrations within appropriate ranges can reduce the risk of vascular calcification. However, this research didn't find a meaningful link between serum calcium or phosphorus levels and CVC in MHD patients. This discrepancy might be due to the widespread use of calcium carbonate and various phosphorus binders at our center in recent years, which could have influenced serum calcium and phosphorus levels. Moreover, single-time-point serum measurements may not fully reflect patients' overall calcium-phosphorus metabolism.

While previous studies have identified dialysis vintage as a risk variable for CVC (11), but this study found that the dialysis age of participants in both groups was similar. This may be due to the small number of participants in this research, all of whom were from a single center.

This study has the following limitations. First, the potential effects of phosphate binders and cholesterol-lowering medications were not adjusted for in the analysis. Patients on phosphate binders may exhibit suppressed serum phosphorus levels due to drug efficacy, potentially obscuring the relationship between phosphorus metabolism and CVC. Similarly, statin use could elevate HDL-C concentrations, which might confound the observed association between HDL-C and CVC risk. Second, the cross-sectional design limits causal inference between VFA and CVC. While associations were identified, temporal relationships and mechanistic pathways require validation through longitudinal studies. Future research should prioritize prospective designs with repeated measurements of VFA and CVC progression, alongside systematic adjustments for medication use (e.g., dose, duration). Additionally, exploring the interplay between visceral adiposity, drug-induced metabolic alterations, and calcification pathways may clarify the biological mechanisms underlying these associations.

In conclusion, elevated VFA is linked to the occurrence of CVC in MHD patients and could potentially act as a biomarker for identifying CVC.

## Data Availability

The original contributions presented in the study are included in the article/Supplementary Material, further inquiries can be directed to the corresponding author.
